# Structure, Luminescence, and Magnetic Properties of Crystalline Manganese Tungstate Doped with Rare Earth Ion

**DOI:** 10.3390/ma14133717

**Published:** 2021-07-02

**Authors:** Jae-Young Jung, Soung-Soo Yi, Dong-Hyun Hwang, Chang-Sik Son

**Affiliations:** Division of Materials Science and Engineering, Silla University, Busan 46958, Korea; ssyi@silla.ac.kr (S.-S.Y.); dhhwang@silla.ac.kr (D.-H.H.); csson@silla.ac.kr (C.-S.S.)

**Keywords:** MnWO_4_, photoluminescence, co-precipitation, magnetic, synthesis

## Abstract

The precursor prepared by co-precipitation method was sintered at various temperatures to synthesize crystalline manganese tungstate (MnWO_4_). Sintered MnWO_4_ showed the best crystallinity at a sintering temperature of 800 °C. Rare earth ion (Dysprosium; Dy^3+^) was added when preparing the precursor to enhance the magnetic and luminescent properties of crystalline MnWO_4_ based on these sintering temperature conditions. As the amount of rare earth ions was changed, the magnetic and luminescent characteristics were enhanced; however, after 0.1 mol.%, the luminescent characteristics decreased due to the concentration quenching phenomenon. In addition, a composite was prepared by mixing MnWO_4_ powder, with enhanced magnetism and luminescence properties due to the addition of dysprosium, with epoxy. To one of the two prepared composites a magnetic field was applied to induce alignment of the MnWO_4_ particles. Aligned particles showed stronger luminescence than the composite sample prepared with unsorted particles. As a result of this, it was suggested that it can be used as phosphor and a photosensitizer by utilizing the magnetic and luminescent properties of the synthesized MnWO_4_ powder with the addition of rare earth ions.

## 1. Introduction

Recently, metal tungstate (MXO_4_, M = Ba, Ca, Mn, Sr, X = Mo, W) has attracted a lot of attention because of its applicability as a multiferroic, light-emitting material, light-emitting diode, and laser [1,2]. Among tungstate materials, the tetragonal scheelite-like structure is a comporting phosphor host material and photocatalytic material because the ${\mathrm{WO}}_{4}^{2-}$ group shows a good absorption rate in the ultraviolet (UV) and blue ranges. This produces a specific emission band through energy transfer from the ${\mathrm{WO}}_{4}^{2-}$ group to the RE ion [3,4,5]. In particular, manganese tungstate (MnWO_4_) crystal is a suitable parent material for doping rare earth and metal ions because of its excellent thermal stability and high energy transfer efficiency from tungsten ions to activator ions; also, rare earth and metal ions are generated by energy transfer between 4f-4f shells [6]. This material has the advantage of generating a high emission intensity with a narrow band gap and a variety of emission wavelengths [7]. The kind and positional symmetry of the activator ions doped in the thermally and chemically stable parent grid are important factors in implementing various types of electrochemical, laser, multiferroic, and display devices [8,9]. The diversity of the MnWO_4_ depends on the type and concentration of activator ions, the sintering temperature, the size of the crystal grain, and the synthesis conditions. Martinez et al. proposed that MWO_4_ (M = Ni, Co, Mn, Cu) can be synthesized using the dissolution-precipitation method and applied to the photocatalytic evaluation field through structural and UV absorbance characteristics analysis [10]. Li et al. synthesized MnWO_4_ nanoparticles using co-precipitation and suggested that electrochemical capacitive performance could be investigated by galvanostatic charge/discharge (GV), cycle electrochemical impedance spectroscopy (EIS), and cyclic voltammetry (CV) [11]. It has been reported that by changing the amount of and type of rare earth ions added by changing the structure and luminescence properties of BaWO_4_, it is possible to synthesize a phosphor with good crystallinity and capable of implementing various colors using the co-precipitation method [10]. In addition, the BaWO_4_:Ln^3+^ (Ln = Eu, Tb, and Dy) powders synthesized via a solid-state reaction, which showed green, yellow, and red emissions [12]. As in the previous literature, various synthesis methods and application cases of tungstate materials have been reported. In this study, the MnWO_4_ precursor was prepared using the co-precipitation method and the optimum synthesis temperature was investigated by varying the sintering temperature. In addition, it was suggested that magnetic and luminescent properties can be realized through the addition of rare earth ions, and that MnWO_4_ can be applied as a fluorescent and photosensitizing material.

## 2. Materials and Methods 

### 2.1. Synthesis of MnWO_4_ and Rare Earth Doped with MnWO_4_

Materials: manganese (Ⅱ) nitrate (Mn(NO_3_)_2_·xH_2_O), sodium tungstate (Na_2_WO_4_), dysprosium (Ⅲ) nitrate (Dy(NO_3_)_3_·xH_2_O, Dy^3+^), and Allied epoxy set were used in this study.

**Figure 1:**
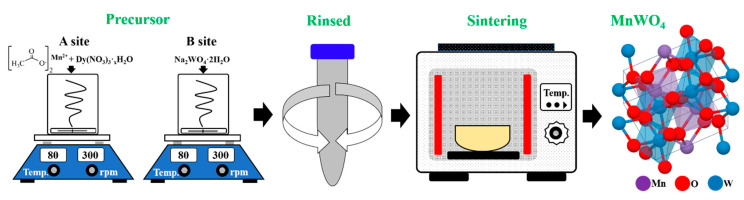
Schematic of MnWO_4_ powder synthesis experimental procedure.

First, two beakers ‘A’ and ‘B’ were prepared. In beaker ‘A’, 1 mmol of Mn(NO_3_)_2_·xH_2_O was added to 50 mL of distilled water (D.I water) and stirred until completely dissolved. In beaker ‘B’, 50 mL of distilled water was added with the same moles of Na_2_WO_4_ and stirred until dissolved. When reagents in both beakers had dissolved, beaker ‘B’ solution was slowly poured into stirring beaker ‘A’ and stirred for about 30 min. After that, powder was obtained using a centrifuge, and the precursor was prepared by rinsing with D.I water twice to remove the remaining sodium. The prepared precursors were dried in an 80 °C oven for about 18 h. The dried precursors were heat-treated at various sintering temperatures (80, 400, 600, 800, 900, and 1000 °C) and then structural characteristics were investigated. In addition, to synthesize MnWO_4_ having magnetic and luminescent properties in the same manner, MnWO_4_:Dy^3+^ powder was synthesized by varying the amount of Dy(NO_3_)_3_·xH_2_O added to beaker ‘A’ when preparing the precursor, as shown in Figure 1.

### 2.2. Chraraterization

The structural properties of the MnWO_4_ and MnWO_4_:Dy^3+^ powders were obtained by X-ray diffraction analysis (XRD; Rigaku Ultima IV, Tokyo, Japan). Raman spectra were obtained using a Raman spectrometer (LabRam-HR 800, Horiba Jobin-Yvon, France), equipped with a 514 nm laser as excitation source. The magnetic properties of the samples were measured using a vibrating sample magnetometer with MnWO_4_ and MnWO_4_:Dy^3+^ samples after magnetization with 6 T pulsing magnetic field. The chemical composition and oxidation state of the synthesized phosphors were investigated by X-ray photoelectron spectroscopy (XPS, ESCALAB 250XI, Waltham, MA, USA). The peak position of the insulating samples was calibrated using a C1 of 285 eV. The surface morphology and microstructure were observed by field emission scanning electron microscopy (FE-SEM, SU-8220, Hitachi, Tokyo, Japan) and transmission electron microscopy (TEM, JEM 2100F, JEOL, Japan). Photoluminescence spectra were obtained through a photomultiplier tube operating at 250 V, a fluorescence spectrophotometer (Scinco, FS-2, Seoul, Korea), and an optical microscope (OM, BX53M, OLYMPUS, Shinjuku, Japan).

### 2.3. Fabrication of MnWO_4_:Dy^3+^ Epoxy Composite 

A composite was prepared by mixing with epoxy to find changes in the luminescence properties of MnWO_4_ particles aligned by magnetic field influence. An epoxy resin and a hardener were prepared at a weight ratio of 10:1, and 3 wt.% of MnWO_4_:Dy^3+^ powder was added and stirred for 1 h. The mixture was poured into a mold and air bubbles were removed in a vacuum desiccator for 1 h. After that, one specimen was hardened as is; the other specimen was hardened by generating a magnetic field by installing magnets on both sides of the mold to align the MnWO_4_:Dy^3+^ particles.

## 3. Results and Discussion

### 3.1. Crystallinity of MnWO_4_ According to Various Sintering Temperatures

**Figure 2:**
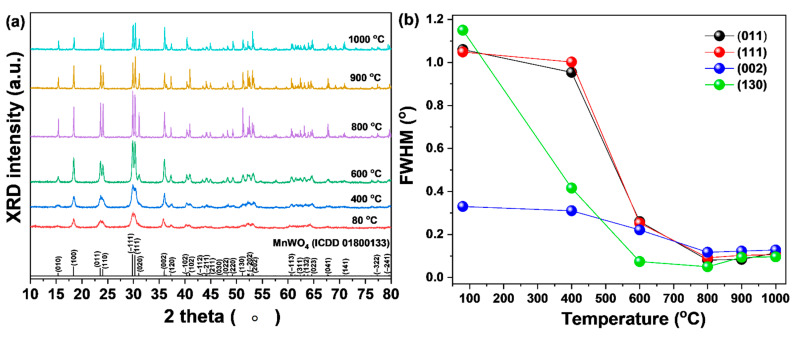
(**a**) XRD patterns of MnWO_4_ and (**b**) FWHM of main peaks according to various sintering temperatures.

The precursor prepared by co-precipitation method was heat-treated at various temperatures to determine the crystallinity and structure of the synthesized MnWO_4_, followed by XRD analysis, with results as shown in Figure 2a. A clear XRD pattern could not be confirmed at relatively low heat treatment temperature, but it was found that MnWO_4_ could be synthesized even at low temperature. At the heat treatment temperature of 600 °C, crystalline MnWO_4_ was confirmed, as in the results of the International Center for Diffraction Data (ICDD 01-080-0133, monoclinic, P2/c) reference. In particular, main peaks of the (111), (011), (002), and (130) phases were identified [13]. In addition, as the sintering temperature increased, the full width at half maximum (FWHM) of the main peaks decreased and showed a tendency to decrease significantly at 800 °C (Figure 2b). As a result of this, it is thought that the increase of the sintering temperature increases the crystallinity of MnWO_4_ [14]. However, there was no significant change after the sintering temperature of 800 °C and, for energy saving, the optimum sintering temperature was determined to be 800 °C. 

### 3.2. Crystallinity, and Magnetic and Chemical State of MnWO_4_ Doped with Dy^3+^ Ions

**Figure 3:**
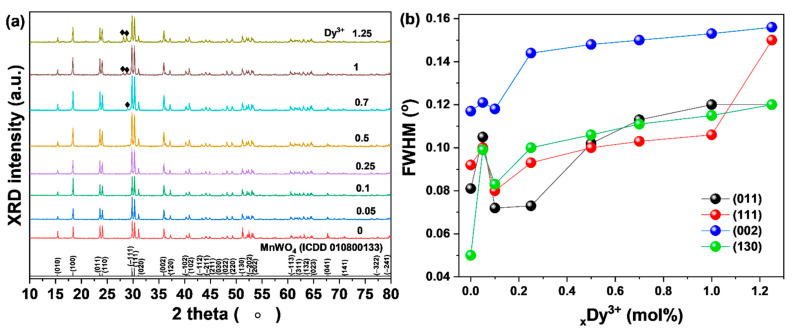
(**a**) XRD patterns of MnWO_4_:Dy^3+^ and (**b**) change of FWHM.

To observe changes in the characteristics of MnWO_4_ with the addition of rare earth materials, precursors were prepared by changing the amounts of dysprosium ions (Dy^3+^; 0.05, 0.1, 0.25, 0.5, 0.7, 1, 1.25 mol.%) added in the process of preparing the precursor by the same experimental method. The prepared precursor was sintered at 800 °C. When the added amount of Dy^3+^ ions was small (0.1 mol.%), the main peaks of the (−111), (011), (002), and (130) phases of the FWHM tended to increase. This is thought to be a phenomenon caused by the addition to the lattice of rare earth ions with relatively large ionic radii. In addition, when the amount of added rare earth material was 0.25 mol.%, the FWHM decreased (Figure 3b), which is thought to have the effect of enhancing the crystallinity [15]. However, as the amount of rare earth ions increased, the FWHM and secondary phase were found. The secondary phase (Figure 3a, black diamond symbol) was identified as a dysprosium oxide phase. When the added amount of rare earth ions was 0.25 mol.% or more, the crystallinity of MnWO_4_ decreased and a secondary phase formed; the critical doping concentration was considered to be 0.25 mol.%. 

**Figure 4:**
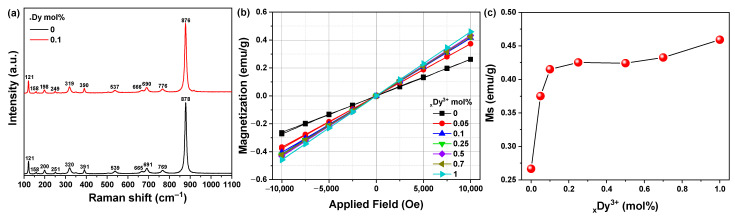
(**a**) Raman shift of MnWO_4_ and MnWO_4_:Dy^3+^, (**b**) magnetization properties of MnWO_4_:Dy^3+^ performed at room temperature, and (**c**) scale of M(10 kOe) vs. Dy^3+^ ions content.

Raman analysis was performed to double check the XRD data obtained for Dy^3+^ ion-doped crystalline MnWO_4_. The vibration bands of MnWO_4_ shown in Figure 4a show six Raman active modes at 200, 320, 391, 539, 691, and 878 cm^−1^, This may be due to the ν(A_g_), r(B_g_), δ(A_g_), symmetric A_g_, (W_2_O_4_)_n_ chain, ν_as_(B_g_) of the Mn cation, and the symmetric A_g_ oscillations of the two terminal WO groups, respectively. There is crystalline MnWO_4_, which correlates accurately with the literature [16]. The very strong band appearing at 878 cm-1 corresponds to the strong symmetrical stretching of the WO_2_ group in MnWO_4_. The bands at 769 and 691 cm^−1^ indicate the existence of weak asymmetric and symmetric tensile vibration modes of W–O–W bonds [17]. The peak at 539 cm^−1^ is attributed to the tensile vibration of Mn–O [18]. The band at 391 cm^−1^ indicates that there is a symmetric stretch of W–O–W [19]. The band at 320 cm^−1^ checked the moderate shear of WO_2_ and W–O–W [20]. The weak vibrating band appears at 251 cm^−1^, indicating the bending mode of [WO_6_]_6_ and the twisting vibrating mode of the WO_2_ group [21]. The two vibrating bands at 158 and 121 cm^−1^ are translational modes of tungsten [22]. In Raman analysis, no significant change was observed between the MnWO_4_:Dy^3+^ and MnWO_4_ samples. To identify changes in the magnetic properties according to the amount of Dy^3+^ added to the synthesized crystalline MnWO_4_, vibrating sample magnetometer (VSM) analysis was performed (Figure 4b).

In addition, it was shown that the magnetic properties increased as the amount of added Dy^3+^ ions increased. Due to its 4f orbital, the Dy^3+^ ion is a prototype of a highly correlated electronic system. It is partially occupied by the f shell, and the 4f orbital is split into seven non-degenerate orbitals. According to Hund’s law, the magnetic moments are caused by the 4f and 5d orbitals, which are consistent with the magnetic moments of the corresponding Dy^3+^ ions [23]. 

**Figure 5:**
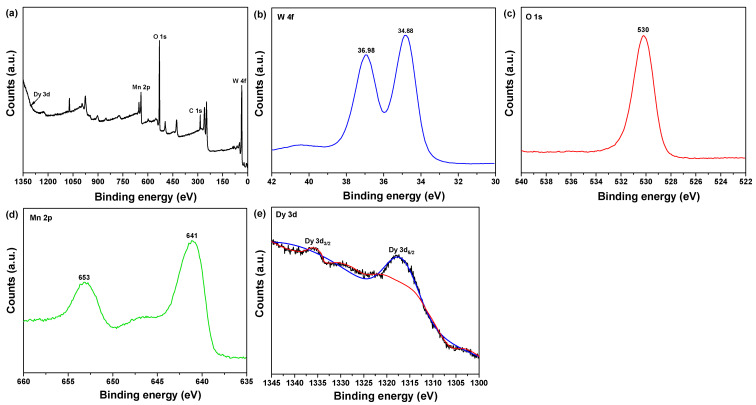
XPS data of MnWO_4_:Dy^3+^: (**a**) survey, (**b**) W 4f, (**c**) O 1s, (**d**) Mn 2p, and (**e**) Dy 3d.

To determine MnWO_4_:Dy^3+^ samples of binding energy, oxidation, and chemical state, XPS analysis was performed on MnWO_4_:Dy^3+^ (Figure 5). Results show Mn 2p; two peaks can be observed corresponding to Mn 2p_3/2_ and Mn 2p_1/2_ at 641 eV and 653 eV, respectively. This indicates that the Mn present in the sample is in +2 oxidation state [24]. The XPS spectrum of W 4f is shown in Figure 5b. It has two peaks, corresponding to W 4f_7/2_ and W 4f_5/2_ at 34.88 and 36.98 eV, respectively. The W 4f_7/2_ and W 4f_5/2_ doublets’ spin-orbit is at 2.1 eV, and the oxidation state of W can be specified as +6 [25]. The O 1s peak shows a main component with a central energy of 530 eV and a lower binding energy, which monitored to the formation of O_2_ oxide-coupled manganese and tungsten elements (Mn–O–W), as shown in Figure 5c. In Figure 5e, the RE^3+^ 3d spectrum can be observed for the MnWO_4_ sample doped with RE^3 +^. The Dy^3+^ 3d spectrum is visible at 1317 eV and 1335 eV; these can be assigned to the RE^3+^ 3d_5/2_ and 3d_3/2_ states, respectively, based on the Dy–O bond [26].

### 3.3. Luminescence and Morphology Properties of MnWO_4_ Doped with Dy^3+^ Ions

**Figure 6:**
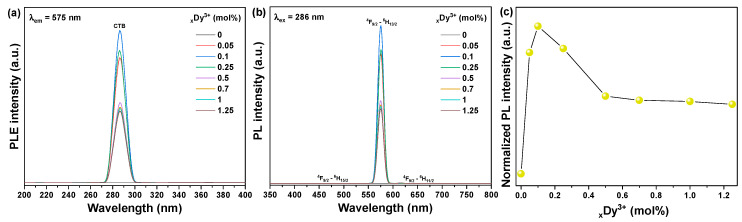
Luminescence properties of MnWO_4_:Dy^3+^: (**a**) PLE spectra under 575 nm, (**b**) PL spectra under 286 nm, and (**c**) integrated PL intensity.

The photoluminescence excitation (PLE) and photoluminescence (PL) spectra of MnWO_4_: Dy^3+^ powder synthesized with activator Dy^3+^ ions of different doping concentrations were obtained. The excitation spectrum monitored at 575 nm consists of peaks in the range of 200 to 400 nm. The PLE spectrum almost covers the ultraviolet region. The excitation band is wide in the range of 270–300 nm to 286 nm, corresponding to the Dy^3+^-O^2−^ charge transfer band (CTB) in the matrix crystal [27,28]. When the concentration of Dy^3+^ increased from 0.05 to 0.25 mol.%, the intensity of all excitation bands increased rapidly, reaching a maximum at 0.1 mol.%, and then decreased significantly within the Dy^3+^ concentration range of 0.5 to 1.25 mol.%, as shown in Figure 6a. For MnWO_4_: Dy^3+^ powder, the emission spectrum under 286 nm excitation shows two main emission bands at 480 and 575 nm, corresponding to the _4_F_9/2_ → _6_H_15/2_ magnetic dipole (MD) transition and the transition of the electric dipole (ED) of _4_F_9/2_ → _6_H_13/2_, respectively. [29] The emission intensity at 575 nm (ED) is very obvious. This result shows that when Dy^3+^ concentration is 0.1 mol.%, the position of the Dy^3+^ ion in the MnWO_4_ host lattice shifts from a non-antisymmetric position to an antisymmetric position. Among all the emission transitions of Dy^3+^, the strongest yellow emission originated from the _4_F_9/2_ → _6_H1_3/2_ ED transition. As the concentration of Dy^3+^ ions increased from 0.5 to 1.25 mol.%, the intensity of the main _4_F_9/2_ → _6_H_13/2_ transition rapidly decreased due to the concentration quenching effect (Figure 6c), mainly due to non-radiant energy transfer between Dy^3+^ activator ions. The critical distance *R_c_* between the Dy^3+^ activator ions can be calculated using the following equation presented by Blasse [30],

(1) $$R_{c}=2{\left(3V/4\pi \;\chi_{c}Z\right)}^{1/3}$$

where *V* is the volume of the unit cell, *X_c_* is the critical concentration (Dy^3+^ ions), and *Z* is the number of host cations in the unit cell. For the MnWO_4_ host, V=146.299 Å^3^, $x_{c}=0.1$ and  Z=2. Therefore, *R_c_* was estimated to be about 11.65 Å. It is well known that there are three types of interactions in which electric multipolar interaction is involved in the energy transfer: dipole–dipole, dipole–quadrupole, and quadrupole–quadrupole interactions. 

**Figure 7:**
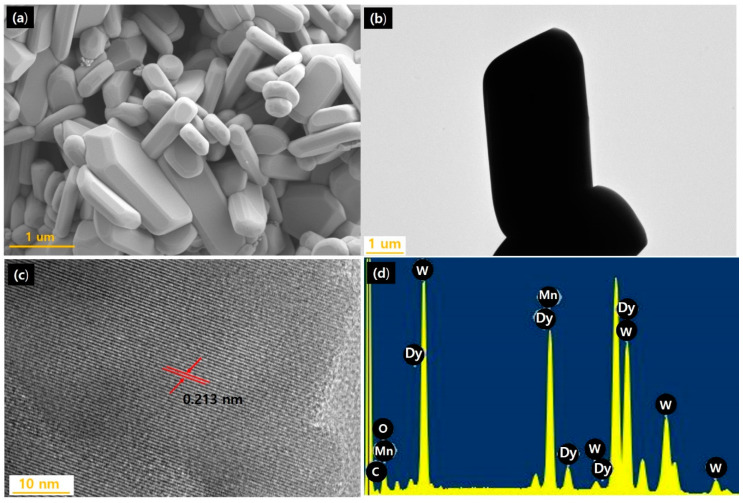
(**a**) SEM image of MnWO_4_:Dy^3+^, (**b**) TEM image of MnWO_4_:Dy^3+^, (**c**) high-resolution image, and (**d**) EDX composition analysis result.

To observe the shape and morphology of the synthesized MnWO_4_:Dy^3+^ particles, FE-SEM and TEM analyses were performed and results are shown in Figure 7. MnWO_4_:Dy^3+^ was generally clustered in the shape of a long, round column in the vertical direction; the size was about 4 µm in length and about 2 µm in width. In high-resolution analysis using TEM, the interplanar distance of the (−111) phase was observed to be about 0.213 nm. This was similar to the value calculated from the XRD data (d_(−111)_ spacing; 0.299 nm). In addition, Mn, W, O, and Dy were detected in EDX component analysis; components of synthesized MnWO_4_:Dy^3+^ were confirmed. 

### 3.4. MnWO_4_:Dy^3+^ Particle Aligned in Epoxy Composite by Magnetic Field 

**Figure 8:**
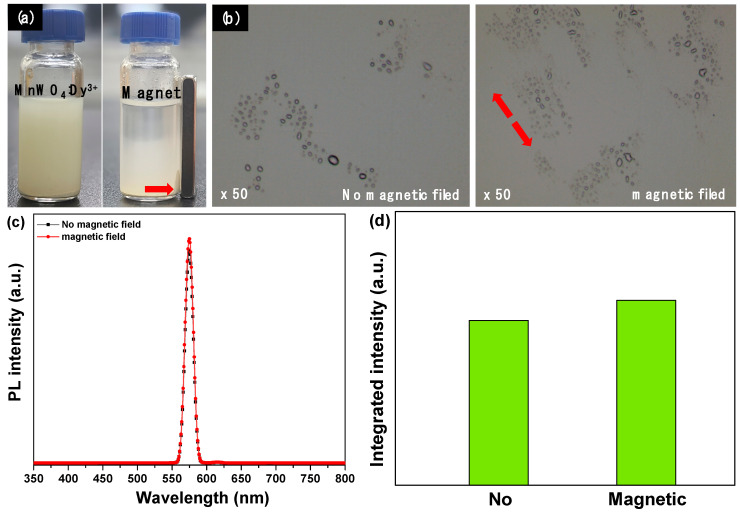
(**a**) Photograph of MnWO_4_:Dy^3+^ powder moved to magnet in ethanol, (**b**) OM images of MnWO_4_:Dy^3+^ particle behavior with magnetic field, (**c**) PL spectra, and (**d**) integrated PL intensity with magnetic field.

As can be seen in Figure 8a, the synthesized MnWO_4_:Dy^3+^ powder was dispersed in ethanol and then a magnet was installed. It was confirmed that the powders moved in the direction of the magnet and can be used as a paramagnetic material. In addition, movements of particles in the mixture made with epoxy polymer were observed through an optical microscope according to the presence or absence of a magnetic field (Figure 8b). MnWO_4_:Dy^3+^ particles were confirmed to align in the direction of the magnetic field. Using these characteristics, we made an epoxy composite and investigated changes in luminescence characteristics according to alignment of particles according to presence or absence of magnetic field (Figure 8c). The composite in which the particles were aligned by exposure to the magnetic field showed a strong luminescence intensity of about 10% (Figure 8d). This is thought to be a phenomenon that occurs because the energy transfer of the aligned particles works slightly more efficiently than in particles in which light energy introduced from the outside is agglomerated. It seems that, utilizing these characteristics, this material can be applied to fields such as display and medical engineering.

## 4. Conclusions

We propose that MnWO_4_ powder synthesis is possible in an easy way. After preparing the MnWO_4_ precursor using the co-precipitation method, the crystallinity change of MnWO_4_ according to various synthesis temperatures was observed. The best crystallinity was exhibited when the heat treatment temperature was 800 °C. At this time, dysprosium, a rare earth ion, was added to enhance light emission and magnetic properties to synthesize MnWO_4_:Dy^3+^. According to the amount of Dy^3+^ ions added, the magnetic properties were enhanced and the luminescence properties were enhanced. When producing an epoxy composite using these properties, it was found that the luminescent properties were enhanced by about 10% as the particles were aligned in the magnetic field direction. It was suggested that the synthesized MnWO_4_:Dy^3+^ powder can be used as a paramagnetic material and can be applied to display and medical industries.

## Data Availability

The data presented in this study are available in the database of the authors at the Faculty of Materials Science and Engineering.
